# Editorial: Aging and health in China

**DOI:** 10.3389/fpubh.2022.998769

**Published:** 2022-09-20

**Authors:** Quanbao Jiang, Qiushi Feng

**Affiliations:** ^1^Institute for Population and Development Studies, Xi'an Jiaotong University, Xi'an, China; ^2^Department of Sociology and Anthropology, Centre for Family and Population Research, National University of Singapore, Singapore, Singapore

**Keywords:** healthy aging, population aging, China, older adults, COVID-19

Population aging is sweeping the globe. In the foreseeable future, though being replaced soon by India as the most populous nation, China is and will be holding the largest older population in the world. According to the most recent census of China in 2020, the proportion of Chinese older individuals aged 65 years old and above has approached nearly 14 percent, suggesting China is becoming an aged society. This is a huge challenge for China nowadays, especially regarding how to meet the health needs of the large size of older Chinese. Research is thus called for, including but not limited to investigations on the prevalence and trend of chronic diseases and their related risk factors, the transformation of eldercare and healthcare, and evaluations of the policies and interventions of population aging. This Research Topic on *Aging and health in China* has collected cutting-edge studies on these critical topics from an interdisciplinary perspective, representing the current research progress in this vital field.

This Research Topic chose three papers under the theme of health trends in China's older population. In “*Trends of healthy life expectancy of the elderly in China in 1994 to 2015: Revisiting from the perspective of morbidity transition*,” Zhang, Dong et al. applied the classic Sullivan method to examine the change in Healthy Life Expectancy (HLE) for the recent two decades, which was measured by activities of daily living (ADL). Interestingly, it was observed that the 1994–2004 period witnessed a trend of morbidity expansion, whereas the 2005–2010 period experienced a scenario of morbidity compression. Yu et al. further explored the trend of hypertension among Chinese older persons in the article “*Hypertension prevalence rates among urban and rural older adults of China, 1991 to 2015: A standardization and decomposition analysis*.” Using nine waves of data from the China Health and Nutrition Survey, the study reported the crude and standardized prevalence of hypertension in both rural and urban China and further examined the temporal changes of the rural-urban gap, which narrowed in the 1993–1997 period, enlarged in the 1997–2011 period, and narrowed again in the 2011–2015 period. Last, Hu, Gu et al. examined the cognitive function trend of Chinese older people in the article “*Trends in cognitive function among Chinese elderly from 1998 to 2018: An age-period-cohort analysis*.” Using eight waves of data from the Chinese Longitudinal Healthy Longevity Survey, the authors found that cognitive function deteriorated with age at an increasing rate, whereas the period effect and cohort effect were relatively small compared to the age effect.

The Research Topic further included four papers to discuss the risk factors for survival, health, and wellbeing of older Chinese. In the article “*Association between functional limitations and incident cardiovascular diseases and all-cause mortality among the middle-aged and older adults in China: A population-based prospective cohort study*,” Hu, Zheng et al. conducted a 7-year longitudinal analysis and found that functional limitations of the middle-aged and older Chinese adults were significantly associated with subsequent cardiovascular diseases and mortality. Along this line of research, Cao et al. further investigated the effect of the sensory impairment on the mortality of older persons in the article “*Is olfactory impairment associated with 10-year mortality mediating by neurodegenerative diseases in older adults? The four-way decomposition analysis*.” Based on the 10-year longitudinal data analysis, they reported that olfactory impairment (OI) was associated with a higher mortality risk amongst older adults, and such an association was stronger with the presence of neurodegenerative diseases (NDDs). Next, Zhang, Xiao et al. examined the patterns and correlates of multimorbidity in later life in the article “*Urban-rural differences in patterns and associated factors of multimorbidity among older adults in China: A cross-sectional study based on apriori algorithm and multinomial logistic regression*.” According to this study, rural older adults had a higher prevalence of multimorbidity than their urban counterparts. It was also found that age, family health history, education, smoking and drinking, and anxiety were significant factors of multimorbidity among older Chinese persons. The last article under this theme, “*Association between self-perceived stigma and quality of life among urban Chinese older adults: The moderating role of attitude toward own aging and traditionality*,” focused on the quality of life. In this article, Sun et al. found that self-perceived stigma contributed to the decreased quality of life among Chinese urban older adults. This association was found to be moderated by the attitude toward own aging and traditionality, the term of which refers to the degree of adherence to traditional values. The study called for more attention to the cultural and ideational factors in understanding the health and wellbeing of older Chinese people.

Negative life events such as widowhood, accident, and disaster have been long documented in the literature to affect health and health behaviors of old ages. Under this theme, this Research Topic had four studies. In “*Negative life events, social ties, and depressive symptoms for older adults in China*,” with the two waves of the Chinese Longitudinal Aging Social Survey, Ruan et al. found a significant association between negative life events and depressive symptoms among Chinese older persons. They further revealed that friendship ties could play a moderating role here, the effect of which was more prominent for male, rural, and less educated individuals. Similarly, in the article “*Stressful life events and Chinese older people depression: Moderating role of social support*,” Yu and Liu confirmed that stressful life events were detrimental to older individuals' psychological health, and this negative association could be buffered by social support. Another two papers under this theme are related to the ongoing COVID-19. In the article “*Lifestyle behaviors and quality of life among older adults after the first wave of the COVID-19 pandemic in Hubei China*,” Duan et al. used the survey data from Hubei province to examine the correlates of the quality of life during the pandemic. They proposed that lifestyle behavior such as physical activity, fruit and vegetable intake, and preventative behaviors such as frequent hand washing, facemask wearing, and social distancing all had significant associations with the quality of life during the pandemic. Chai further examined how the COVID-19 outbreak affected older adults' health literacy and health behavior in the article “*How has the nationwide public health emergency of the COVID-19 pandemic affected older Chinese adults' health literacy, health behaviors and practices, and social connectedness? Qualitative evidence from urban China*.” Based on a series of qualitative interviews, the authors reported that during the COVID-19 period, older Chinese developed effective coping strategies, in which information and communication technology (ICT) played a significant role in obtaining information and maintaining social connections.

In China, social support from spouses and children is essential for the health of older people, especially those from rural regions where the social security system is yet underdeveloped. For this critical theme, this Research Topic presents four articles. In the article “*Widowhood and health status among Chinese older adults: The mediation effects of different types of support*,” Guo et al. examined the health effect of widowhood and the role of support. They found that the negative effect of widowhood on health was partially attributable to the lack of emotional support and companionship after losing a spouse. Emotional support and companionship, as revealed, mediated the association with mental health and physical health, respectively, but such mediations were found to be significant only among women and among rural residents. In the article “*The health effect of the number of children on Chinese elders: An analysis based on Hukou category*,” Long et al. found that older Chinese with only one child had poorer health than those with multiple children, though this association was not statistically significant for urban older adults. Furthermore, the authors proposed some potential mechanisms, such as intergenerational exchange, as explanations. Zhang and Harper further differentiated social support between sons and daughters in the article “*Son or daughter care in relation to self-reported health outcomes for older adults in China*.” With data from the three waves of the China Health and Retirement Longitudinal Study, they found that the Chinese older parents reported better health if being cared for by sons than by daughters, and this disparity was more remarkable in rural areas, for older mothers, and in poorer families. Last, focusing on the oldest old Chinese, Zeng et al. reported in the article “*The expected demand for elderly care services and anticipated living arrangements among the oldest old in China based on the Andersen model*” that coresidence with children is still the most preferred living arrangement of Chinese older people. This study also revealed a significant gap in meeting the health need of the Chinese oldest old people.

Lastly, this Research Topic included a few studies with significant policy and intervention relevance. For instance, in the article “*Development and implementation of couple-based collaborative management model of type 2 diabetes mellitus for community-dwelling Chinese older adults: A pilot randomized trial*,” Liu et al. introduced the new model of diabetes management, which highlights the need to mobilize the family members and has a good potential of applications. Next, as smart health technology is recently proposed as an effective measure to maintain and promote the health and wellbeing of older people, we included the article “*Adoption intention and factors influencing the use of gerontechnology in Chinese community-dwelling older adults: A mixed-methods study*.” In this paper, Huang et al. reported that most Chinese older adults had intentions of adopting these smart health technologies, though their intentions varied. Under this theme, we also had two papers on the health expenditure of older Chinese. In the article “*Catastrophic health expenditure associated with frailty in community-dwelling Chinese older adults: A prospective cohort analysis*,” Fan et al. found that frailty was a significant predictor of catastrophic health expenditure (CHE) in Chinese older people. As CHS may be associated with the excessive financial burden on households and even lead to poverty, this paper called for interventions against frailty in old age. Moreover, Gao et al., in the article entitled “*Forecasting the health transition and medical expenditure of the future elderly in China: A longitudinal study based on Markov chain and two part model*,” divided the self-rated health into different statuses, calculated age-specific transition probabilities across these statuses, estimated status-specific individual medical expenditure, and finally projected the future medical expenses of older adults in China from 2020 to 2035. Finally, in the article “*The multidimensional relative poverty of rural older adults in China and the effect of the health poverty alleviation policy*,” Zeng et al. constructed the multidimensional relative poverty index (MRPI), in which health was considered a critical component. Through an empirical evaluation of the health poverty alleviation policy in rural areas of Shaanxi China, the authors validated the use of MRPI and further proposed that health promotion programs for older people, such as the introduction of basic medical insurance and long-term care insurance, should be integrated into the campaigns against poverty in China.

As introduced above, this Research Topic covers exciting topics and presents important findings on the health and wellbeing of older Chinese people. The included studies illustrate the broad scope of this field and its practical values and make up a good collection of academic work to promote healthy aging in China. The complicated context of China, we consider, may further add value to these studies in the general literature of public health and gerontology. The health and wellbeing of Chinese older adults are often affected by major social transformations and events in recent years, such as the ongoing epidemiological transition, rapid-changing households and living arrangements, large-scale rural/urban migration, economic and technological development, expanding social security system, as well as COVID-19 outbreaks. Studies in this Research Topic have addressed some of these issues above, but more work is demanded to follow up with this important line of research. Since the Chinese Government has recently announced the proactive response to population aging as the national prioritized policy rationale, we think now is never a better time to promote studies on healthy aging in China, with the sincere hope that this Research Topic could be a part of this grand mission.

## Author contributions

Both authors listed have made a substantial, direct, and intellectual contribution to the work and approved it for publication.

## Conflict of interest

The authors declare that the research was conducted in the absence of any commercial or financial relationships that could be construed as a potential conflict of interest.

## Publisher's note

All claims expressed in this article are solely those of the authors and do not necessarily represent those of their affiliated organizations, or those of the publisher, the editors and the reviewers. Any product that may be evaluated in this article, or claim that may be made by its manufacturer, is not guaranteed or endorsed by the publisher.

